# How stroke-related dysphagia relates to quality of life: the mediating role of nutritional status and psychological disorders, and the moderating effect of enteral nutrition mode

**DOI:** 10.3389/fnut.2024.1339694

**Published:** 2024-03-14

**Authors:** Hongji Zeng, Xi Zeng, Nana Xiong, Lichun Wang, Ying Yang, Liugen Wang, Heping Li, Weijia Zhao

**Affiliations:** ^1^School of Public Health, Zhengzhou University, Zhengzhou, China; ^2^Department of Rehabilitation Medicine, The First Affiliated Hospital of Zhengzhou University, Zhengzhou, China; ^3^Peking University Sixth Hospital, Beijing, China; ^4^NHC Key Laboratory of Mental Health (Peking University), Beijing, China; ^5^National Clinical Research Centre for Mental Disorders (Peking University Sixth Hospital), Beijing, China; ^6^Cangzhou Hospital of Integrated Chinese and Western Medicine, Hebei, Cangzhou, China; ^7^Department of Rehabilitation Medicine, The People’s Hospital of Suzhou New District, Suzhou, China

**Keywords:** stroke, dysphagia, quality of life, nutritional status, psychological disorders, enteral nutrition, intermittent oro-esophageal tube feeding

## Abstract

**Background:**

Although stroke-related dysphagia has been shown to influence quality of life (QOL), the underlying mechanisms have yet to be uncovered.

**Objective:**

This study aims to investigate the mediating role of nutritional status and psychological disorders in the relationship between stroke-related dysphagia and QOL in stroke patients and explore the moderating effect of enteral nutrition mode.

**Methods:**

In 2022, A questionnaire survey using stratified random sampling was conducted on 5,322 stroke patients with dysphagia, including Functional Oral Intake Scale (FOIS), Swallowing Quality of Life Questionnaire, Patient Health Questionnaire-9 (PHQ-9), and Generalized Anxiety Disorder-7 (GAD-7) to assess dysphagia, QOL and psychological disorders, respectively, for each participant. Records of serum albumin, Hemoglobin, Total serum protein, serum prealbumin and Body mass index were enrolled to assess nutritional status.

**Results:**

FOIS demonstrated a significant positive predictive effect on QOL. Nutritional status and psychological disorders (PHQ-9 and GAD-7) mediated the relationship between FOIS and QOL. Nutritional status-psychological disorders showed a chain mediation effect in the relationship between FOIS and QOL. The moderating effect of enteral nutrition mode was observed.

**Conclusion:**

The mediating role of nutritional status and psychological disorders with moderating effect of enteral nutrition mode in the relationship between dysphagia and QOL in stroke patients was found.

## 1 Introduction

Stroke is a leading cause of disability and death globally, with approximately 600,000 new cases reported each year in the United States and approximately 17.88 million stroke patients have been reported in China (1, 2). Neurological impairments resulting from stroke, including decreased muscle strength and coordination, negative mental conditions, and limited verbal abilities, significantly impact patients’ quality of life (QOL) (3, 4). With global aging, stroke has emerged as a major public health threat (5). Dysphagia, one of the most frequent and potential complications of stroke, occurs in 37–78% of cases and is associated with pneumonia and malnutrition (6). Based on comorbidity mechanisms, the interplay among various complications often leads to a vicious cycle, further exacerbating the condition of patients (7). Therefore, understanding the mechanisms by which stroke-related dysphagia is related to QOL holds significance for improving the prognosis and easing the burden of the disease.

Stroke-related dysphagia has gained considerable attention and numerous studies have demonstrated its statistical association with QOL (8). However, the existing literature has mainly focused on clinical observations and regression analyses, emphasizing the effect of different therapies on dysphagia and analyzing related factors (9). Previous research has shown that QOL in stroke patients with dysphagia is influenced by both psychological and physiological factors (3). Nutritional status is essential for stroke patients’ recovery and is a vital indicator to assess the value of different nutrition support modes (10). Additionally, post-stroke depression and anxiety are common mental health issues, which were frequently chosen as manifest variables of psychological disorders (11). On the other hand, dysphagia has been recognized to have social, physiological, psychological, and other attributes that can impact patients’ overall health from various aspects (12). Currently, these indicators have been frequently adopted by studies applying various interventions to such patients. Nevertheless, the specific underlying mechanisms are often discussed theoretically without sufficient empirical research (13). Furthermore, in mainland China, due to the low acceptance of gastrostomy, Intermittent Oro-esophageal Tube Feeding (IOE) and Nasogastric Tube Feeding (NGT) are the primary choices to alleviate dysphagia-related malnutrition (14). Although both two enteral nutrition modes have demonstrated therapeutic effects, it remains unclear how they impact the relationship between dysphagia and QOL (15).

**Figure 1:**
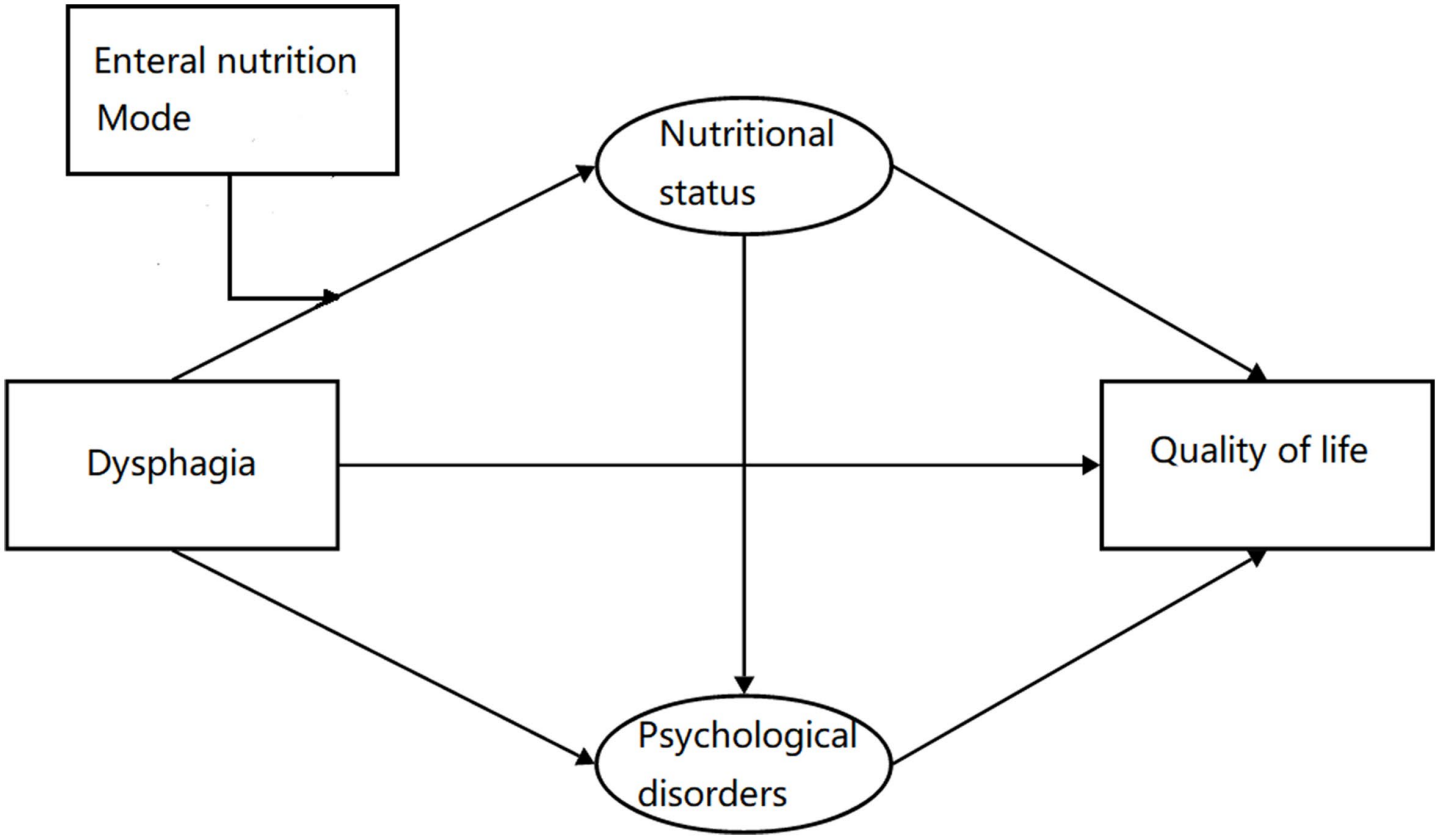
Hypothetic model.

Due to the complexity of the disease mechanism, the relationships between various factors and QOL are not simply linear (16). Inadequate research into the precise mechanisms can result in insufficient targeted treatment, thereby diminishing therapeutic efficacy. Therefore, based on the biopsychosocial model, this study aimed to investigate the impact of dysphagia on nutritional status and psychological disorders, ultimately extending to the overall QOL outcome (17). Firstly, we hypothesized that nutritional status and psychological disorders would act as mediators in the relationship between dysphagia and QOL, both independently and interactively. Secondly, we proposed that the association between dysphagia and nutritional status was influenced by the moderating effect of enteral nutrition mode. Building upon these hypotheses, our objective was to elucidate the underlying mechanisms through which stroke-related dysphagia influenced QOL (Figure 1). By doing so, we aimed to provide theoretical foundations and empirical support for improving QOL and developing effective rehabilitation intervention strategies for stroke patients with dysphagia.

## 2 Methods

### 2.1 Procedures and study participants

This was a stratified random sampling, cross-sectional study conducted from January 2022 to December 2022. We divided China into five major regions: eastern China, western China, southern China, northern China, and central China. In each region, one primary hospital, one secondary hospital, and one tertiary hospital were randomly selected and included in the study. Data were collected from the patients and their respective hospitals using questionnaires and medical records. The invited participants were informed of the topic prior to their tentative consent to complete the survey. After the review by the Medical Ethics Committee of the First Affiliated Hospital of Zhengzhou University in 2021, the content and process of the project complied with the international and national ethical requirements for biomedical research, and all methods were carried out in accordance with relevant guidelines and regulations (ethics number: KY-2021-0096-2). Inclusion criteria were as follows: (1) age ≥ 18 years; (2) diagnosed with stroke; (3) confirmed with dysphagia by flexible endoscopic evaluation of swallowing or video fluoroscopic swallowing study and receiving enteral nutrition support through NGT or IOE for at least 15 days consecutively; (4) able and willing to provide information needed; and (5) course of disease less than 28 days. The exclusion criteria were as follows: (1) complicated with other neurological diseases, hematologic diseases, hepatic failure, renal failure, or tumors; (2) tracheostomy was conducted during hospitalization; and (3) complicated with other diseases that could cause dysphagia.

### 2.2 Assessment

#### 2.2.1 Dysphagia

Dysphagia was assessed using the Functional Oral Intake Scale (FOIS), according to which dysphagia was divided into seven levels as follows: Level 1, inability to take food via oral; Level 2, severe tube feeding dependence; Level 3, moderate tube feeding dependence; Level 4, oral intake with limited restrictions; Level 5, able to take food via oral with strict limitations on food consistency; Level 6, able to take food via oral with mild limitations on food consistency; and Level 7, normal oral intake without any restrictions (18). In the current study, Cronbach’s α coefficient for this scale was 0.902.

#### 2.2.2 QOL

The Swallowing Quality of Life questionnaire (SWAL-QOL) was used to evaluate QOL, including 44 items from 11 main domains influenced by dysphagia, using a Likert-5 point scoring system. The final scores were converted to a standard percentage scale from a maximum total score of 220 (19). Cronbach’s α coefficient for the SWAL-QOL scale in the current study was 0.906, while the test–retest reliability was 0.847.

#### 2.2.3 Nutritional status

The latest test results for albumin (ALB, g/L), hemoglobin (Hb, g/L), total protein (TP, g/L), prealbumin (PA, mg/L), and body mass index (BMI, Kg/m^2^) were recorded on the day of the survey. Patients who did not have a test result available within 72 h prior to our investigation were asked to undergo another test. Patients who refused would be excluded from the study.

#### 2.2.4 Psychological disorders

Psychological disorders were divided into depression and anxiety. The former was assessed using the Patient Health Questionnaire-9 (PHQ-9), with aspects including mood swings, optimism, sleep quality, appetite, etc. The total PHQ-9 score ranges from 0 to 27, and is positively correlated with potential depression (20). In this study, the PHQ-9 demonstrated a Cronbach’s α coefficient of 0.913. The Generalized Anxiety Disorder-7 (GAD-7) was used to assess participants’ anxiety, including items related to fear, worry, attention, etc. The total score ranges from 0 to 21, and is positively correlated with potential anxiety (21). In this study, the GAD-7 demonstrated a Cronbach’s α coefficient of 0.879.

#### 2.2.5 Enteral nutrition mode

The enteral nutrition mode for participants was classified into two categories based on self-report or medical records: (1) NGT and (2) IOE. Specifically, NGT is performed according to relevant guidelines by nursing staff, who insert the tube through the nasal cavity into the stomach and secure it to the face. The IOE was performed by professionals who inserted the tube through the mouth into the upper end of the esophagus and removed it after each feeding. The recommended feeding volume of IOE is advised not to exceed 500 mL (22).

#### 2.2.6 Control variables

The control variables in this study included the type of permanent residence (rural or urban) and gender.

### 2.3 Statistical analysis

Continuous variables were presented as mean ± standard deviation ( $\bar{x}$ ±s), while categorical variables were presented as the number of cases and percentages (n, %). Harman’s single-factor test was conducted to test common method bias. Pearson analysis was used to analyze the correlation between continuous variables, while a *t*-test was performed to analyze the differences between continuous variables under group. The 95% bias-corrected bootstrap confidence intervals (CIs) of the indirect effects were calculated based on 5,000 bootstrap samples. Mediation analysis was conducted using Mplus 8.0, and moderated mediation models were examined using Process 3.3 in SPSS 21.0. A difference was considered statistically significant if the *p* value was ≤0.05.

## 3 Results

A total of 6,517 patients were initially enrolled in this study. After excluding incomplete or unreliable data, 5,322 patients were finally enrolled, of which 58.08% were male. Among these participants, 2,456 (46.15%) were from rural areas, and 2,866 (53.85%) were from urban areas. The average age of the participants was 54.74 years old, with a standard deviation of 5.94.

### 3.1 Test of common method bias

To examine the presence of common method bias, Harman’s single-factor test was conducted as some of the data were derived from patient self-reports. The results showed that out of the 11 factor eigenvalues, the first factor explained 19.24% of the variance (<40%), indicating the absence of significant common method bias in the current study.

### 3.2 Descriptive statistics and correlation analysis

After testing for normality and homogeneity of variance, the t-test was found to be applicable. The results of the correlation analysis demonstrated a significant negative correlation between QOL and psychological disorders (PHQ-9 and GAD-7) and a significant positive correlation between QOL and dysphagia (assessed using FOIS) and nutritional status. It is also found that dysphagia was significantly negative correlated with psychological disorders, and significantly positive correlated with nutritional status. Additionally, a significant negative correlation was observed between nutritional status and psychological disorders. IOE was reported as enteral nutrition mode by 1,477 participants (27.75%) and NGT by 3,845, and there were significant differences in dysphagia, QOL, nutritional status, and psychological disorders among patients who underwent IOE vs. NGT therapy. The details were shown as Table 1.

**Table 1:** Result of descriptive statistics and correlation analysis (*N* = 5,322).

Variables	Mean	Standard deviation	1	2	3	4	5
1. QOL	49.73	9.74	1				
2. Dysphagia	3.74	2.01	0.532*	1			
3. Nutritional status	83.12	11.59	0.374*	0.287*	1		
4. Psychological disorders	12.36	4.17	−0.477*	−0.29*5	−0.208*	1	
5. Enteral nutrition mode	N/A	N/A	2.501*	1.996*	2.771*	−2.096*	1

*p < 0.05.

### 3.3 Mediating model

**Figure 2:**
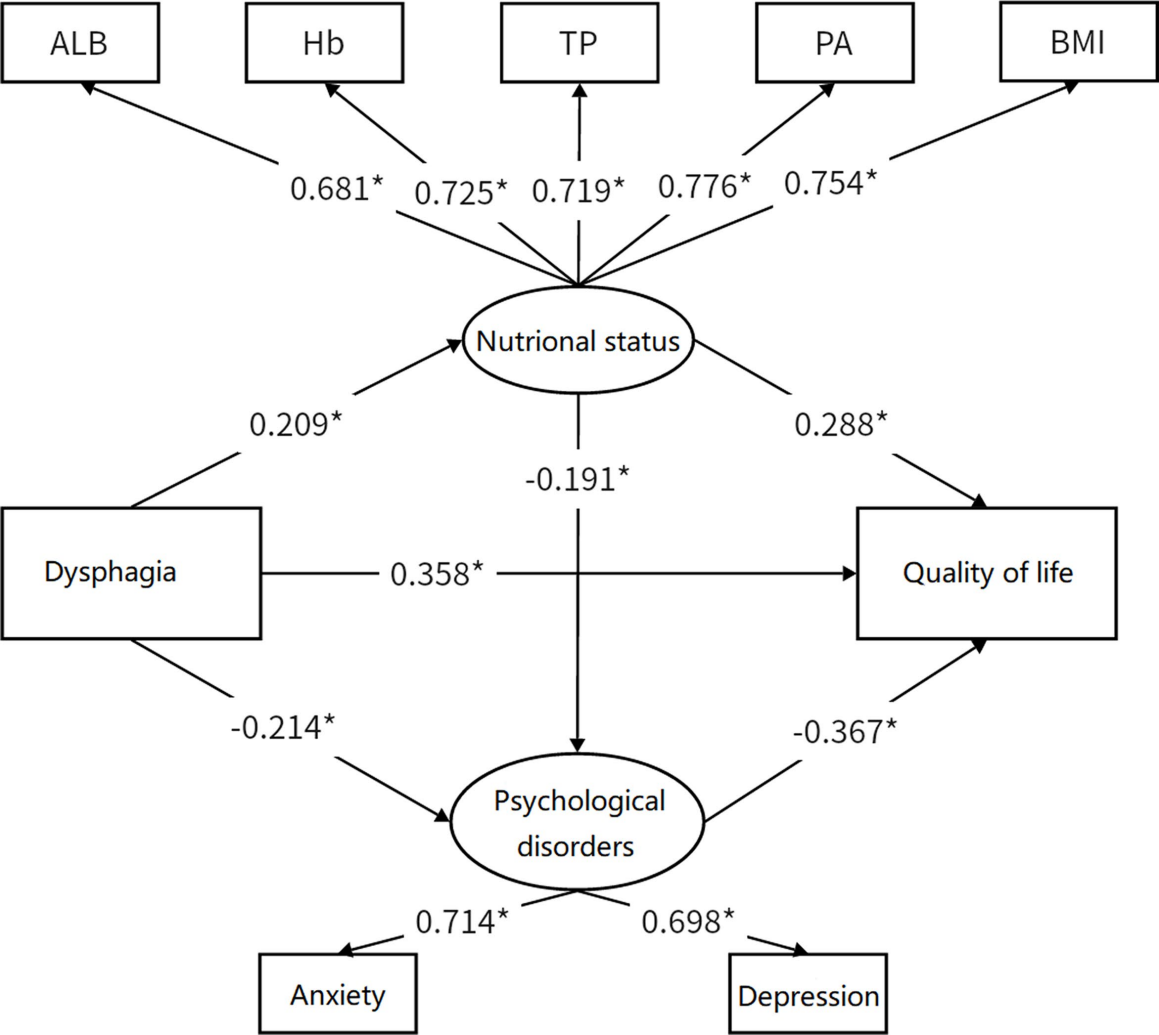
Mediation model. ^*^p < 0.05, Standardized regression coefficients were demonstrated on path. Coefficients of control variable have been omitted for simplicity of the model.

After controlling for gender and type of permanent residence, a mediation model was used to analyze the mediating effects, as shown in Figure 2. After examination, all factor loadings were found to be greater than 0.6, indicating an excellent model fit: CFI = 0.961, TLI = 0.942, SRMR = 0.030, RMSEA = 0.053 (95% CI: 0.047–0.058). The results showed that compared to urban participants, rural participants expressed lower QOL (*β* = 0.049, *p* < 0.05), while female participants expressed higher QOL than males (*β* = 0.032, *p* < 0.05). Among all patients, direct effects (*β* = 0.358, 95% CI: 0.305–0.411, *p* < 0.001) and indirect effects (*β* = 0.154, 95% CI: 0.103–0.205, *p* < 0.001) were found for dysphagia on QOL. Nutritional status (*β* = 0.060, 95% CI: 0.048–0.072, *p* < 0.001) and psychological disorders (*β* = 0.079, 95% CI: 0.059–0.099, *p* < 0.001) partially mediated the relationship between dysphagia and QOL. Besides, nutritional status and status-psychological disorders (*β* = 0.015, 95% CI: 0.007–0.022, *p* < 0.001) showed a chain mediation effect. The total effect of dysphagia on QOL was 0.512 (95% CI, 0.462–0.562), and the mediating effect accounted for 30.08% of the total effect.

### 3.4 Moderated mediation model

Participants were divided into two groups with different enteral nutrition modes to further examine the moderating effect on the relationship between dysphagia and QOL: the IOE group (with IOE therapy) and the NGT group (with NGT therapy). The details were presented in Table 2. In this table, sections with no results were replaced with dashes, indicating that no results were obtained either because, according to the model, the independent variables should not have an impact on the predictor variable in the corresponding equation, or because a variable should not have an impact on itself.

**Table 2:** Testing for moderated mediation.

Equation I				Equation II			Equation III		
Predictor variables: Psychological disorders				Predictor variables: Nutritional status			Predictor variables: QOL		
	*β*	*t*	95% CI	*β*	*t*	95% CI	*β*	*t*	95% CI
Dysphagia (X)	−0.262*	−7.172	−0.298 – −0.226	0.206*	2.743	0.177–0.235	0.375*	16.689	0.323–0.426
Nutritional status (M1)	−0.197*	−2.581	0.176–0.218	–	–	–	0.293*	9.414	0.248–0.338
Psychological disorders (M2)	–	–	–	–	–	–	−0.334*	−5.284	−0.392 – −0.276
Enteral nutrition mode (W)	–	–	–	0.451*	11.489	0.378–0.584	–	–	–
Interaction term (X*W)	–	–	–	0.132*	4.791	0.058–0.206	–	–	–
*R*^2^	0.242			0.146			0.281		

All variables have been standardized, *p < 0.05.

**Figure 3:**
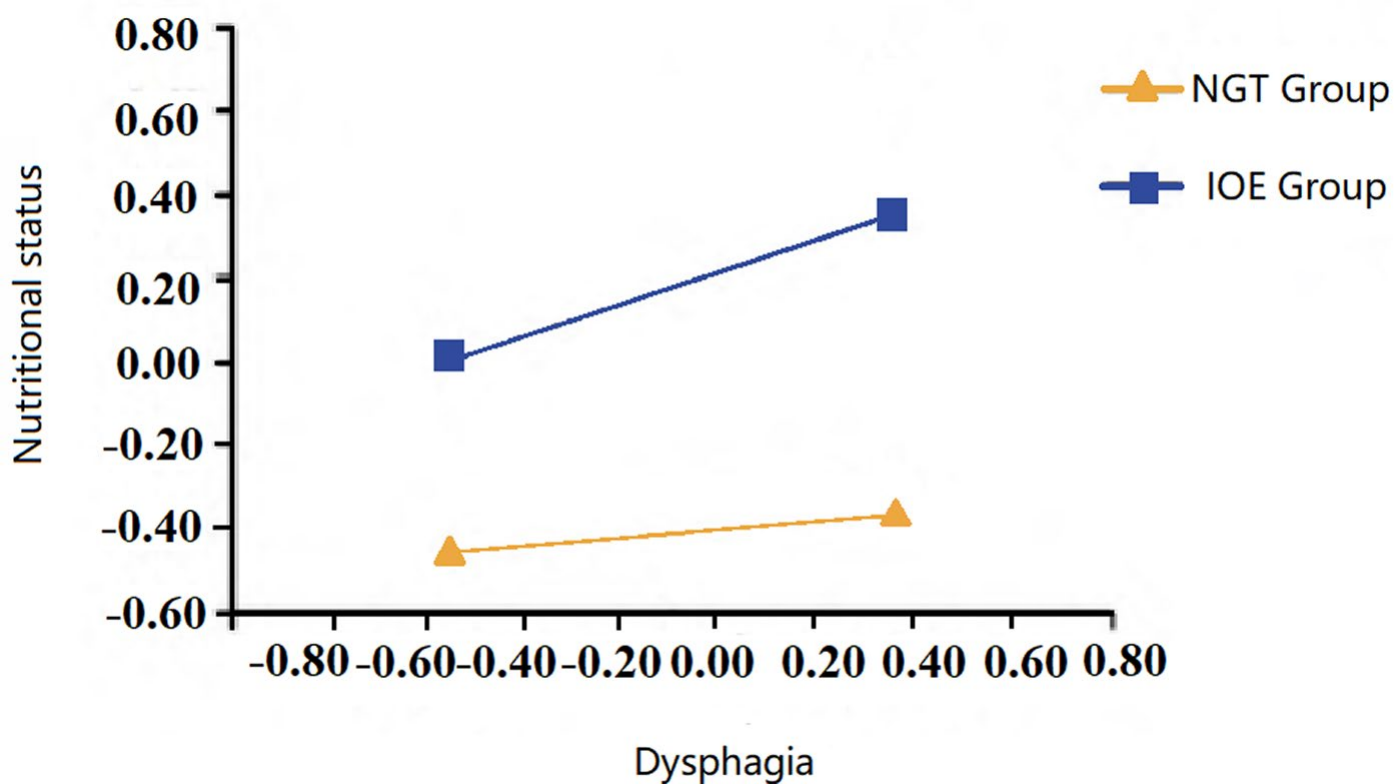
Moderating effect of eternal nutrition method on the relationship between dysphagia and nutritional status. Coefficients of control variable have been omitted for simplicity of the model.

Equation II revealed that dysphagia, IOE therapy, and their interaction term all had a significant positive effect on predicting nutritional status. Through slope testing, it was found that when the NGT was used, dysphagia had a significant positive predictive effect on nutritional status (*β* = 0.09, SE = 0.05, *p* < 0.05). When IOE was used, the positive predictive effect of dysphagia on nutritional status was significantly enhanced (*β* = 0.31, SE = 0.03, *p* < 0.001), indicating that the enteral nutrition mode moderates the impact of dysphagia on nutritional status, as shown in Figure 3.

## 4 Discussion

Stroke is a prevalent disease worldwide, and dysphagia, a common complication of stroke, is significantly associated with a reduced QOL (23). Our study found that female stroke patients had better QOL than males, and urban participants had better QOL compared to their rural counterparts, consistent with prior research findings (24, 25). Under these circumstances, investigating the impact of stroke-related dysphagia on QOL and its underlying mechanisms is of great practical significance for healthy aging and patient well-being.

### 4.1 The relationship between dysphagia and QOL

The current study revealed a significant positive predictive role of dysphagia on QOL, consistent with previous research findings (12). To understand these potential mechanisms, it is important to consider how dysphagia affects eating experiences and life satisfaction. Factors such as slow or discontinuous meal experience and limited dietary choices due to dysphagia can have a negative impact on the overall QOL (26). Moreover, dysphagia can prolong illness and lead to repeated hospitalizations due to complications such as aspiration, reflux pneumonia, and vomiting. These events not only disrupt income and time management but can also result in disability, further reducing quality of life (27). Additionally, eating holds social significance in human society beyond its nutritional intake. Dysphagia can hinder patients from participating in various social activities, including banquets, thereby negatively affecting their quality of life (28). Therefore, the improvement of dysphagia should be emphasized. Swallowing-related rehabilitation training should be promoted and actively utilized according to individual circumstances. In addition, healthcare providers should regularly track patients’ rehabilitation progress and comprehensively monitor improvements in swallowing function, to adjust the rehabilitation intervention plan as needed.

### 4.2 Mediating role of nutritional status

This study revealed that dysphagia can indirectly impact quality of life through its effect on nutritional status. Dysphagia can lead to inadequate chewing and moistening of food in the oral cavity, dependence on tube feeding, and even gastrointestinal disorders, all of which can hinder digestion and absorption (29). Stroke patients in the rehabilitation period typically experience elevated levels of inflammation and high metabolic demands. An inferior nutritional status may result in weight loss, muscle weakness, and exacerbation of functional impairment, ultimately affecting rehabilitation outcomes (30). Additionally, stroke patients have already been prone to immune dysfunction, and inferior nutritional status can further compromise their immunity, worsening their overall health condition (31). Malnutrition can also contribute to reduced concentration, cognitive decline, limited mobility, and fatigue, ultimately resulting in negative subjective experiences, hindering social participation, and ultimately impairing quality of life (32). Therefore, in addressing dysphagic stroke patients, emphasis should be placed on nutritional support and management. This includes providing appropriate dietary plans to ensure patients receive sufficient nutrients, promoting recovery, and reducing the occurrence of complications. Additionally, regular assessment and monitoring of patients’ nutritional status should be conducted, with adjustments made as necessary.

### 4.3 Mediating role of psychological disorders

Stroke-related depression is a well-known issue that can significantly impact stroke patients’ prognosis (33). The current study demonstrated that psychological disorders played a mediating role in the association between dysphagia and QOL. There are several potential explanations for this association. Dysphagia is easily perceived by patients due to its related symptoms, which can undermine their confidence in recovery (16). Additionally, patients may actively or subconsciously avoid social activities because of dysphagia, leading to feelings of loneliness and psychological disorders including depression. This can trigger inflammation in the body, exacerbate neurovascular damage, and further negatively affect recovery and prognosis (34). Moreover, psychological disorders can cause an imbalance of neurotransmitters in the brain, such as serotonin and dopamine, which affects normal communication and functional regulation between brain regions, resulting in cognitive and behavioral disorders that ultimately lower quality of life (35). Furthermore, psychological disorders are associated with a decrease in stroke patients’ compliance with rehabilitation and treatment, further affecting prognosis and quality of life. It is important to address psychological disorders in stroke patients as they can have a significant impact on their overall recovery and quality of life (36). Psychosocial interventions such as cognitive-behavioral therapy and group therapy have been shown to be effective in improving psychological outcomes and reducing depression in patients with stroke (37). Therefore, psychological intervention should be integrated as part of comprehensive treatment. By addressing the psychological needs of patients with stroke, healthcare professionals can improve their overall recovery and QOL.

### 4.4 The moderating effect of enteral nutrition mode

Generally, enteral nutrition supports are used in clinical practice for both safety and nutritional reasons in patients with stroke. The current study revealed that the choice of enteral nutrition mode can moderate the relationship between dysphagia and QOL. Specifically, compared with NGT, patients using IOE demonstrated better nutritional status, indicating that IOE can help mitigate the negative impact of dysphagia on nutritional status. Several factors may contribute to these findings. Firstly, compared to NGT, IOE allows patients to consume a larger volume of nutrients at a time without the risk of reflux, as it does not interfere with the normal function of the cardia (38). This enables IOE to better meet the nutritional needs of patients and to reduce the risk of reflux-related complications. Second, long-term indwelling of NGT may lead to persistent foreign body stimulation and inflammation, which can hinder nutrient absorption and even cause low-grade fever. In contrast, IOE can be easily removed after feeding, without affecting digestion and absorption, potentially leading to better nutritional outcomes (39). Lastly, clinical observations suggest that NGT use may lead to complications such as gastrointestinal bleeding and dysfunction, whereas IOE has demonstrated lower stimulation and fewer side effects (15). Additionally, IOE conforms to the natural physiological structure of the human body, helping to maintain normal gastrointestinal function and effectively improve nutritional status (40). Therefore, in the absence of relevant contraindications, when considering enteral nutrition, the introduction of IOE can be prioritized for patients. Subsequently, the choice of nutritional support mode should be determined through shared decision-making between doctors and patients, rather than the uniform NGT treatment.

### 4.5 Strengths and weaknesses of the study

Based on previous research, the current study conducted a large-sample multicenter investigation to explore the mechanisms underlying the impact of dysphagia on QOL in stroke patients. Additionally, this study further investigated the moderating effect of enteral nutrition mode. This study aimed to elucidate the relationships among frequent outcomes adopted in related research and to provide references for improving the QOL of these patients. However, there are some limitations to this study. Firstly, although this investigation revealed the relationships among the variables above, difficulties exist for the cross-sectional design to infer causal relationships. In fact, psychological disorders may also have a reverse effect on nutritional status, suggesting the existence of bidirectional causality. Therefore, in the future, tracking designs and randomized interventions should be incorporated to further explore causal relationships. Lastly, the number of variables included in this study was relatively limited. Most importantly, due to the extremely limited number of patients with gastric fistula in mainland China, we were unable to include them in the comparison. In the future, we plan to expand our research scope to include other countries and incorporate more variables and cases to ensure the representativeness and comprehensiveness of the study.

## 5 Conclusion

The current study revealed that: (1) Dysphagia demonstrated a significant positive predictive effect on QOL; (2) Nutritional status and psychological disorders mediated the relationship between dysphagia and QOL; (3) Nutritional status-psychological disorders showed a chain mediation effect in the relationship between dysphagia and QOL; and (4) The moderating effect of enteral nutrition mode was observed, with IOE enhancing the positive effects of dysphagia on nutritional status.

## Data availability statement

The raw data supporting the conclusions of this article will be made available by the authors, without undue reservation. Because the data is not attached to the appendix or the paper itself but is also available from the corresponding author.

## Ethics statement

The studies involving humans were approved by after review by the Medical Ethics Committee of the First Affiliated hospital of Zhengzhou University in 2021, the content and process of the project complied with the international and national ethical requirements for biomedical research and all methods were carried out in accordance with relevant guidelines and regulations (Ethic number: KY-2021-0096-2). The patients/participants provided written informed consent to participate in this study. The studies were conducted in accordance with the local legislation and institutional requirements.

## Author contributions

HZ: Conceptualization, Data curation, Formal analysis, Investigation, Software, Visualization, Writing – original draft. XZ: Conceptualization, Funding acquisition, Investigation, Methodology, Project administration, Resources, Supervision, Validation, Writing – review & editing. NX: Conceptualization, Data curation, Formal analysis, Investigation, Methodology, Supervision, Writing – review & editing. LicW: Conceptualization, Data curation, Investigation, Resources, Supervision, Validation, Writing – review & editing. YY: Conceptualization, Data curation, Investigation, Resources, Supervision, Validation, Writing – review & editing. LiuW: Conceptualization, Data curation, Investigation, Supervision, Validation, Writing – review & editing. HL: Conceptualization, Data curation, Investigation, Supervision, Validation, Writing – review & editing. WZ: Conceptualization, Data curation, Formal analysis, Investigation, Software, Writing – review & editing.
